# Saliva as a Candidate for COVID-19 Diagnostic Testing: A Meta-Analysis

**DOI:** 10.3389/fmed.2020.00465

**Published:** 2020-08-04

**Authors:** László Márk Czumbel, Szabolcs Kiss, Nelli Farkas, Iván Mandel, Anita Hegyi, Ákos Nagy, Zsolt Lohinai, Zsolt Szakács, Péter Hegyi, Martin C. Steward, Gábor Varga

**Affiliations:** ^1^Department of Oral Biology, Faculty of Dentistry, Semmelweis University, Budapest, Hungary; ^2^Institute for Translational Medicine, Medical School, University of Pécs, Pécs, Hungary; ^3^Doctoral School of Clinical Medicine, University of Szeged, Szeged, Hungary; ^4^Department of Dentistry, Oral and Maxillofacial Surgery, Medical School, University of Pécs, Pécs, Hungary; ^5^Department of Conservative Dentistry, Faculty of Dentistry, Semmelweis University, Budapest, Hungary; ^6^School of Medical Sciences, University of Manchester, Manchester, United Kingdom

**Keywords:** coronavirus, SARS-CoV-2, COVID-19, diagnostic tests, saliva, systematic review, meta-analysis

## Abstract

**Background:** COVID-19 is a serious and potentially deadly disease. Early diagnosis of infected individuals will play an important role in stopping its further escalation. The present gold standard for sampling is the nasopharyngeal swab method. However, several recent papers suggested that saliva-based testing is a promising alternative that could simplify and accelerate COVID-19 diagnosis.

**Objectives:** Our aim was to conduct a meta-analysis on the reliability and consistency of SARS-CoV-2 viral RNA detection in saliva specimens.

**Methods:** We have reported our meta-analysis according to the Cochrane Handbook. We searched the Cochrane Library, Embase, Pubmed, Scopus, Web of Science and clinical trial registries for eligible studies published between 1 January and 25 April 2020. The number of positive tests and the total number of tests conducted were collected as raw data. The proportion of positive tests in the pooled data were calculated by score confidence-interval estimation with the Freeman–Tukey transformation. Heterogeneity was assessed using the *I*^2^ measure and the χ^2^-test.

**Results:** The systematic search revealed 96 records after removal of duplicates. Twenty-six records were included for qualitative analysis and 5 records for quantitative synthesis. We found 91% (CI 80–99%) sensitivity for saliva tests and 98% (CI 89–100%) sensitivity for nasopharyngeal swab (NPS) tests in previously confirmed COVID-19 patients, with moderate heterogeneity among the studies. Additionally, we identified 18 registered, ongoing clinical trials of saliva-based tests for detection of the virus.

**Conclusion:** Saliva tests offer a promising alternative to NPS for COVID-19 diagnosis. However, further diagnostic accuracy studies are needed to improve their specificity and sensitivity.

## Introduction

COVID-19, caused by the SARS-CoV-2 virus, is a serious and potentially deadly disease. Globally, as of 5 May 2020, there have been 3,489,053 confirmed cases of COVID-19 reported to WHO, including 241,559 deaths (1). Early diagnosis and isolation of infected individuals will play an vital role in stopping the further escalation of the pandemic.

At present, nasopharyngeal swabbing, followed by reverse transcription of the extracted RNA and quantitative PCR (RT-qPCR), is the gold standard for detection of SARS-CoV-2 infection (2). Specimen collection currently requires trained medical personnel (3), thus exposing staff to a high risk of infection (4). These tests are not always successful at the first attempt, and shortages of swabs and protective equipment are frequently reported (2). Additionally, mass testing requires an increased number of trained personnel at specimen acquisition sites. Consequently, the nasopharyngeal swab (NPS) collection method is causing an economic and logistic burden on healthcare systems. Additionally, nasopharyngeal swabbing causes discomfort to the patients (5) and there are several contraindications, such as coagulopathy or anticoagulant therapy, and significant nasal septum deviation (6). Clearly, there is a need for a simpler and less invasive method that also reduces the risk to healthcare personnel.

One candidate for non-invasive specimen collection is saliva. The saliva secreted by salivary glands contains water, electrolytes, mucus, and digestive and protective proteins (7–9). But whole saliva collected from the mouth is a mixture of glandular secretions, gingival crevicular fluid, serum, expectorated airway surface liquid and mucus, epithelial and immune cells from the oral mucosa and upper airways, and oral microbes and viruses (10). Despite its heterogeneous origins, this mixed fluid is used widely and successfully as a diagnostic tool to identify various oral and systemic conditions (8, 11). These already include viral infections such as dengue, West Nile, chikungunya, Ebola, Zika and Yellow Fever, and also the recently emerged coronaviruses responsible for severe acute respiratory syndrome (SARS) and Middle East respiratory syndrome (MERS) (12).

Since early January 2020, several papers have been published on the possible use of saliva as a specimen for detecting SARS-CoV-2 in the diagnosis of COVID-19. Until now there has been no systematic review or meta-analysis of this topic. Our aim, therefore, was to conduct a meta-analysis, thus overcoming the limitations of the small sample sizes in individual studies, in order to estimate the diagnostic sensitivity of saliva-based detection of the virus. We also aimed to summarize the study protocols that have been registered in clinical trial registries to investigate saliva-based COVID-19 diagnosis in the future.

## Materials and Methods

### Protocol and Registration

The reporting of our meta-analysis follows the guidelines of the Preferred Reporting Items for Systematic Reviews and Meta-Analyses (PRISMA) (13). The PRISMA checklist for our work is available in the supporting information (Table S1). We registered our meta-analysis protocol in the OSF (Open Science Framework, Center for Open Science) registries on 23 April 2020 (https://osf.io/3ajy7).

#### Deviation From the Registered Protocol

Studies eligible according to our inclusion criteria did not present sufficient raw data to complete 2 × 2 contingency tables. True positive, true negative, false positive and false negative values were not generally available, thus sensitivity and specificity could not be separately calculated. Instead, positive event rates were pooled for statistical analysis. Details of the analysis are described in section Summary Measures and Synthesis of Results.

### Eligibility Criteria

We included records if they met the following eligibility criteria: (1) records published in scientific journals or clinical trial registries; (2) patients diagnosed with COVID-19; (3) index test: saliva specimens with PCR diagnostics for detecting SARS-CoV-2; (4) reference standard (comparator test): NPS specimens with PCR diagnostics for detecting SARS-CoV-2; (5) records written in English or available in English translation. Exclusion criteria: (1) publications with no primary results such as reviews, guidelines and recommendations; (2) publications dated before 1 January and after 25 April, 2020; (3) gray and black literature.

### Search Strategy

Systematic searches for records published in English after 1 January 2020 were performed in five major literature databases (Cochrane Library, Embase, PubMed, Scopus, Web of Science) and also in five clinical trial registers (ClinicalTrial.gov, EU Clinical Trials Register, NIPH Clinical Trial Search, ISRCTN Registry, ANZCTR Registry). The last update of our systematic search was performed on 25 April 2020. Cited and citing papers of the relevant studies were screened for further eligible studies.

The following key words were applied to each database to identify eligible records: (COVID 19 OR COVID19 OR Wuhan virus OR Wuhan coronavirus OR coronavirus OR 2019 nCoV OR 2019nCoV OR 2019-nCoV OR SARS CoV-2 OR SARS-CoV-2 OR NCP OR novel coronavirus pneumonia OR 2019 novel coronavirus OR new coronavirus) AND (saliva).

### Study Selection

We used EndNote X9.3.3 reference manger to organize records. After removal of duplicates, two authors (A.H. and I.M.) independently screened the records for eligibility based on the titles and abstracts. Papers included at this stage were further appraised by reading the full text. Any disagreement between reviewers was resolved by consulting a third reviewer (L.M.C.).

### Data Collection

Using a preconstructed, standardized data extraction form, two authors (A.H. and I.M.) independently collected data from the included records. From primary studies the following information was extracted (Table 1): first author's name, year of publication, place of study, study type, population size, age, gender, method of diagnosis, type of PCR kit, and the following outcome parameters: numbers of total, positive and negative saliva tests and numbers of total, positive and negative NPS tests. From registered study protocols the following information was extracted (Table S2): clinical trial ID, recruiting status, study type, number of centers, study design, location, population, intervention, comparison, primary outcomes, and secondary outcomes. In cases of disagreement during extractions a third author (L.M.C.) was consulted.

**Table 1:** Summary of study characteristics of included records.

**References**	**Country**	**Study type**	**Population**		**Diagnoses of COVID-19**	**PCR kit**	**Reference standard**	**Index test**	**Outcome parameters**
			**n (m/f)**	**Age**					
(14)	Italy	Consecutive case series	25 (17/8)	61 (mean) (39–85)	Viral RNA detection with PCR from NPS	Luna Universal qPCR Master Mix	NPS	Saliva	Number of positive and negative index tests
(15)	South Korea	Consecutive case series	4 (2/2)	61.5 (35–82)	Viral RNA detection with PCR from NPS And clinical signs of pneumonia	N/A	NPS	Saliva	Number of positive and negative index tests
(16)	China	Consecutive case series	32 (16/16)	41 (34–54)	Viral RNA detection with PCR from NPS	N/A	NPS	Saliva	Number of positive and negative index tests
(17)	Hong Kong, China	Consecutive case series	23 (13/10)	62 (37–75)	Viral RNA detection with PCR from NPS	QuantiNova Probe RT-PCR Kit	NPS	Saliva	Number of positive and negative index tests
(18)	Australia	Consecutive case series	39 (not published)	Not published	Viral RNA detection with PCR from NPS	Coronavirus Typing (835 well) assay	NPS	Saliva	Number of positive and negative index tests
**Not included in quantitative synthesis:**									
(19)	China	Case report	1 (0/1)	39	Viral RNA detection with PCR from NPS And clinical signs of pneumonia	N/A	NPS	Saliva	Number of positive and negative reference tests and index tests
(20)	South Korea	Case report	1 (0/1)	Neonate (27 day-old)	Viral RNA detection with PCR from NPS	PowerChek TM 2019-nCoV Real-time PCR Kit	NPS	Saliva	Number of positive and negative reference tests and index tests
(21)	USA	Consecutive case series	29 (16/13)	59 (mean) (23–91)	Viral RNA detection with PCR from NPS	The US CDC real-time RT-PCR primer/probe sets	NPS	Saliva	Number of positive and negative reference tests and index tests

*NPS, Nasopharyngeal swab; N/A, Not available*.

### Risk of Bias and Applicability Assessment

We evaluated the potential for bias, the quality of reporting and the applicability of the studies using the QUADAS-2 tool (Quality Assessment of Diagnostic Accuracy Studies 2) (22), which is a tool widely used to assess studies of diagnostic accuracy. Our appraisal consisted of evaluating the risk of bias and applicability in four domains: (1) patient selection, (2) conduct and interpretation of the index test, (3) reference standard, and (4) flow and timing. We applied the following review question to judge the applicability of the studies to our investigation: *Are saliva specimens reliable for detecting SARS-CoV-2 in COVID-19 patients confirmed by nasopharyngeal swab testing?*

We used the preconstructed form available on the QUADAS-2 web page of the University of Bristol (23).

### Summary Measures and Synthesis of Results

In the synthesis of quantitative data we included patient-based data from consecutive case series. Case reports from single participants were excluded.

The sensitivities of the saliva and NPS tests were assessed in patients who had previously been confirmed to be infected, having had both a positive NPS test and well-defined clinical symptoms on admission to the hospital. Extracted data were limited to test results from subsequent occasions when both saliva and NPS samples were collected concurrently. Therefore, the sensitivity of the NPS test is based on the matching NPS tests when saliva tests were also performed.

The sensitivity of the saliva test in the patient-based pooled data was calculated using the methods recommended by the working group of the Cochrane Collaboration. Because some of the sensitivity values are close to or equal to 1, the score confidence interval estimation (24) was applied with the Freeman–Tukey double arcsine transformation (25). Because of the variability of the population sizes and methodologies in the different studies, the DerSimonian and Laird method (26) was used, with 95% confidence intervals (CI), for a random-effects meta-analysis.

Heterogeneity was assessed using the *I*^2^ measure and the χ^2^-test, where *p* < 0.1 is taken to indicate significant heterogeneity. *I*^2^ values of 25, 50, and 75% were identified as low, moderate and high estimates, respectively (27). Statistical analyses were carried out using STATA software version 15.0 (STATACorp, Texas, USA).

## Results

### Study Selection

**Figure 1:**
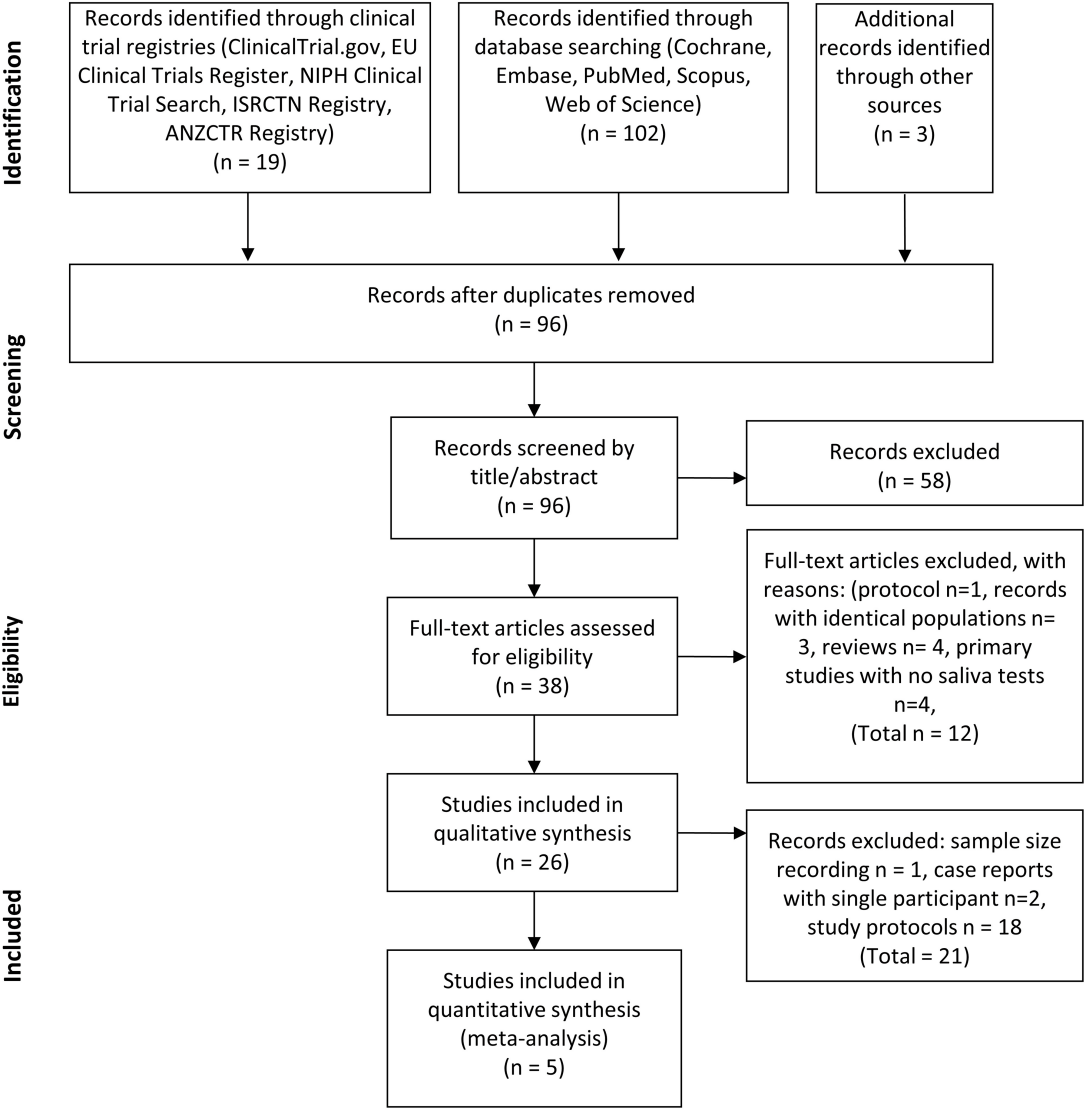
PRISMA flow diagram of the study selection process. Flow chart illustrating the selection process for identifying eligible records.

We included 20 articles for full-text evaluation of completed studies. Of these, eight were included in the qualitative synthesis, from which five were also included in the quantitative synthesis. Figure 1 illustrates the study selection process.

Our search in the clinical trial register yielded 19 protocols, of which one was excluded due to its relating to a different topic.

### Study Characteristics

#### Characteristics of the Studies Included

All five records included in the quantitative synthesis were consecutive case series, involving 123 patients from five distinct global locations (Table 1) (14–18). All of these publications included patients with confirmed diagnoses of COVID-19. No other restrictions on inclusion were stated in any of the studies.

In the qualitative synthesis we also included another consecutive case series (Table 1). But in their work Wyllie et al. presented 38 matching NPS and saliva samples from 29 patients without identifying the double or multiple samplings from individual patients. Therefore, their sample-wise results cannot be combined for quantitative analysis with the others which reported patient-wise data (21).

### Results of Individual Studies and Synthesis of Results

#### Diagnostic Potential of Saliva Specimens

In the individual studies included in the quantitative synthesis, the sensitivity of the saliva test among COVID-19 infected patients ranged from 78% (16) to 100% (14).

**Figure 2:**
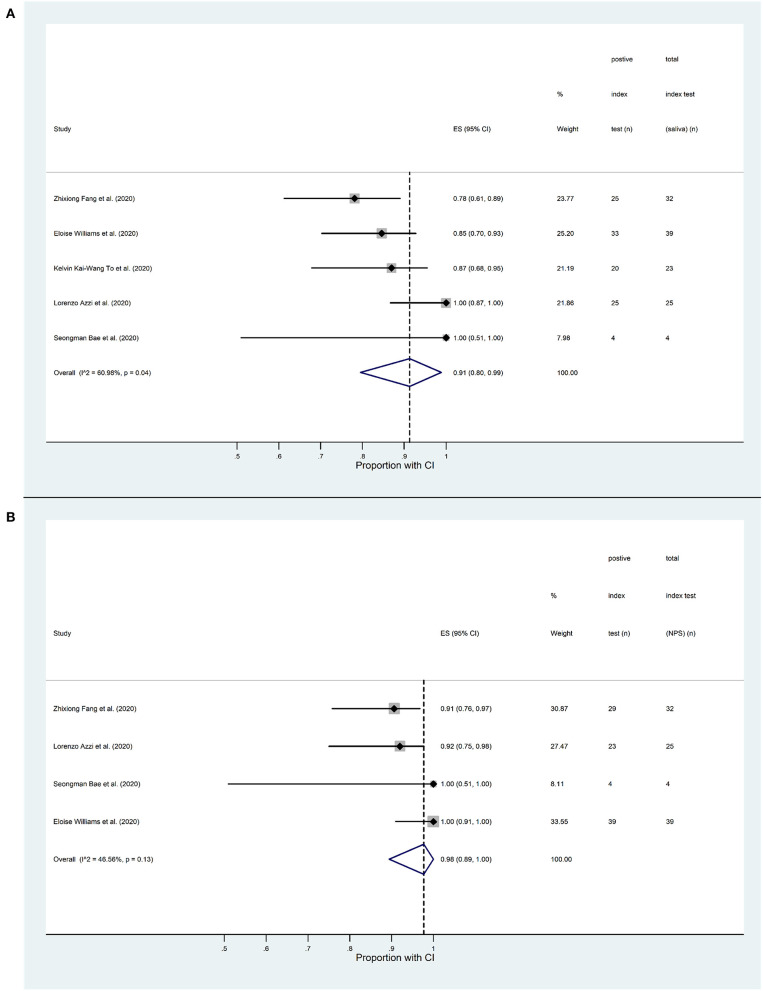
Forest plot analysis of SARS-CoV-2 detection sensitivity based on RT-qPCR analysis of saliva and nasopharyngeal swab (NPS) specimens from COVID-19 patients. **(A)** Proportion of positive saliva tests in the five studies included in the quantitative analysis, ranging from 0.78 to 1. The overall proportion in the pooled data is 0.91 (CI 0.80–0.99). The *I*^2^ value (60.98%, *p* = 0.04) indicates a moderate level of statistical heterogeneity. **(B)** Proportion of positive NPS tests in the four studies included in the quantitative analysis, ranging from 0.91 to 1. The overall proportion in the pooled data is 0.98 (CI 0.89-1). The *I*^2^ value (46.56%, *p* = 0.13) indicates a low level of statistical heterogeneity.

Pooled event rates (positive and negative test results) from saliva specimens show that the sensitivity of the saliva test was 91% (CI 80–99%) among COVID-19 patients diagnosed in the recruitment period (Figure 2A). By definition, the nature of the initial diagnosis implies or rather assumes a 100% sensitivity for the nasal swab test in those patients at that time point. However, pooled event rates from NPS specimens taken concurrently with the saliva specimen collections, generally some time after the initial diagnosis, indicate that the sensitivity of the NPS test, based on these time-matched samples, was 98% (CI 89–100%) (Figure 2B). Since the two confidence intervals overlap, it appears that the proportions of positive test results from the saliva and NPS samples are not very different. However, a firm conclusion will require formal diagnostic accuracy tests based upon larger clinical studies.

We assessed our pooled results for inconsistency using the *I*^2^-test (28). In the case of the saliva tests we found a moderate level of heterogeneity (*I*^2^ = 60.98%, *p* = 0.04) indicating the contribution of confounding factors. On the other hand, we found a low level of heterogeneity among the NPS test results (*I*^2^ = 46.56%, *p* = 0.13).

Interestingly some of the data suggest that NPS tests may occasionally be negative when the corresponding saliva test gives a positive result. Azzi et al. reported that two patients showed positive saliva tests while their NPS tests were negative (14), and a case report showed that in seven sample pairs from one individual, the NPS tests were all negative while the saliva tests were positive on each occasion (19). In a sample-based study of 38 patients, Wyllie et al. (21) detected SARS-CoV-2 in saliva but not NPS specimens from eight patients (21%), while the virus was detected in NPS but not saliva in only 3 matched samples (8%). And overall, they found significantly higher SARS-CoV-2 titers in the saliva than in the NPS specimens.

In a more detailed study, Bae et al. examined the difference in viral loads between the two sampling methods: the values were 0.06 to 3.39 log_10_ units higher in the NPS specimens than in the saliva specimens (15). One case series (18) and another case report on a 27-day-old neonate (20) also found that there were higher viral loads in the NPS specimens.

Only two studies assessed the specificity of the saliva tests (18, 21). In one, a subset of saliva specimens from 50 patients with PCR-negative nasal swabs was tested. SARS-CoV-2 was detected in 2% (CI 0.1–11.5%) of these saliva samples (18). The other study tested 98 asymptomatic healthcare workers with parallel NPS and saliva tests. NPS tests turned out to be negative for all participants, while saliva tests were positive for two (21).

### Risk of Bias Within Studies

We assessed the risk of bias in the six included case series (14–18, 21) according to the QUADAS-2 tool. Five of the six (14–17, 21) had low risk of selection bias. On the other hand four studies (14–17) had high risk of bias in the index test due to the fact that the saliva tests results were interpreted with prior knowledge of the results of the reference standard. Flow and timing were high or unclear in all studies, since there was no exact information regarding the time passed between specimen collections for the two tests. Applicability had low concerns in index test in four studies (14, 17, 18, 21) and unclear in two studies (15, 16). A summary of the risk-of-bias analysis and applicability concerns is available in Tables S3, S4. Altogether, our risk-of-bias analyses demonstrated a moderate bias level in both the individual and the overall aspects of the studies.

#### Ongoing Registered Clinical Trials on Saliva Diagnostics for COVID-19

We also systematically searched five clinical trial registers (EU Register, ISRCTN, ANZCTR, JPRN, ClinicalTrials.gov) for clinical trial protocols that are planned to evaluate saliva specimens for COVID-19 diagnosis. By using the same keywords as for the studies already completed, we found 18 registered clinical trials on planned or ongoing clinical studies. All of them appeared in the ClinicalTrials.gov registry (Table S2). Among these, 13 are non-interventional, focusing primarily on the diagnostic and prognostic value of various specimens collected from patients, including NPS, saliva and blood, in detecting and following the progression of COVID-19 disease. The other five, interventional studies are examining the effectiveness of several potentially beneficial compounds, including azithromycin, lopinavir/ritonavir, beta-cyclodextrin, citrox 3 and peginterferon lambda, on the outcomes of SARS-CoV-2 infection. In these studies, besides NPS specimen collections, saliva tests are also planned. Unfortunately, in the trial protocols very little information is available about the optimization and validation of the saliva collection protocols, the transportation and storage of the saliva samples, the viral RNA assay methods to be used for the saliva samples, and the choice of appropriate internal controls, which is important given the scarcity of human DNA in saliva samples.

## Discussion

In April 2020 the Food and Drug Administration (FDA) granted emergency use authorization (EUA) to Rutgers' RUCDR Infinite Biologics and its collaborators for a new specimen collection approach that utilizes saliva as the primary test biomaterial for the SARS-CoV-2 coronavirus, the first such approval granted by the federal agency (https://www.fda.gov/media/136877/download). This new saliva-based diagnostic collection method, which RUCDR has developed in partnership with Spectrum Solutions and Accurate Diagnostic Labs (ADL), claims to allow an easier and therefore broader screening of the population compared with the current method using nose and throat swabs. Another accelerated EUA for the “Curative-Korva SARS-Cov-2 Assay,” which was specifically designed for use with oral fluid samples, was also approved to permit the testing of oral fluids, i.e., saliva (https://www.fda.gov/media/137088/download). Nasopharyngeal swabs, oropharyngeal swabs and nasal swabs can also be used with the Curative-Korva SARS-CoV-2 Assay, but their performance with this assay has not yet been assessed (https://www.fda.gov/media/137088/download). These two saliva-based, FDA-approved assays are now in use to test for COVID-19 infection, in spite of the fact that no independent, scientific analysis has yet established their effectiveness. Our present work is the first integrative meta-analysis study to review the existing multi-study evidence for validity of the saliva-based approach.

The use of saliva as a diagnostic tool for various systemic conditions is nothing new. Considerable research effort has been made in the past to seek biomarkers in saliva, since its collection is non-invasive and easy. As a result, emerging evidence indicates that whole saliva can be used to identify various oral and systemic conditions [for reviews see (8, 11, 29)]. Importantly, the concept of using saliva to detect viral infections is now well-established (12, 30).

Among RNA viruses, salivary diagnostic tests for Zika are well-established (31, 32) and a number of salivary-based detection methods have been reported for Ebola virus detection (12). The presence of considerable quantities of viral RNA in the saliva of 17 SARS-infected patients has also been shown unequivocally (33). But most studies lack any direct comparison of the sensitivity and specificity of NPS- and saliva-based assays. The one important exception is a study which compared saliva and NPS specimens for the detection of respiratory viruses by multiplex RT-PCR (4). This study, which included results from 236 patients with 11 different viral respiratory infections, including coronaviruses, revealed no significant difference in the sensitivity and specificity of saliva- and NPS-based tests (4). Taken together, although saliva-based diagnostics are supported by a considerable amount of evidence, routine applications are still rare because of the lack of well-standardized protocols.

The source of SARS-CoV-2 in saliva is unknown at present but it could come from multiple locations. One obvious source is debris from the nasopharyngeal epithelium which drains into the oral cavity (17). Secondly, SARS-CoV-2 may actually infect the salivary glands and the virus is then secreted into the saliva from the glands. No information is available on this. But it is of note that during the infection of rhesus macaques by the SARS coronavirus, epithelial cells lining salivary gland ducts are an early target of the virus (34). One consequence of this is the production of SARS-specific secretory immunoglobulin A into the saliva (35). Thirdly, SARS-CoV-2 from blood plasma may access the mouth via the crevicular fluid, an exudate derived from periodontal tissues (36). Fourthly, infected oral mucosal endothelial cells, which show overexpression of ACE2 during SARS-CoV-2 infection, may also contribute to the viral load in saliva (37). Finally, salivary cells may endocytose viruses and virus-containing exosomes from the circulation at their basolateral surface and release them into the salivary lumen by exocytosis. Such mechanisms have been revealed for other macromolecular constituents of the blood, such as DNA and RNA (8). Any or all of these five possible sources may contribute to the appearance of SARS-CoV-2 in the saliva of COVID-19 patients. Given also that the main sites of viral infection (nasal, oral, pharyngeal or respiratory tract) may differ between individuals, it is quite possible that in some patients the virus is more readily detected in the saliva and in others it is more readily detected in an NPS specimen. Such differences might also be related to genomic variations between patients (38). Consequently discrepancies between NPS and saliva test results, rather than indicating a deficiency in one or other test, may be an expected outcome, and it may have implications in terms of assessing asymptomatic carriers (39, 40). Either way, our present level of understanding paves the way for more intensive studies of these important issues, extending well-beyond the design of better diagnostics for SARS-CoV-2 infection (6, 38).

In the present meta-analysis we found that the test sensitivities for SARS-CoV-2 were 91% (CI 80–99%) and 98% (CI 89–100%) for saliva and for NPS samples, respectively, based the pooled event rates among COVID-19 patients. Clearly the two confidence intervals overlap, suggesting that the outcomes of the saliva tests and NPS tests are not very different. There appears to be a slight tendency for NPS tests to be more sensitive but this is not statistically significant. On the other hand, one study reported the opposite tendency with the virus detectable in the saliva but not the NPS sample on a significant number of occasions (21). Although NPS-based SARS-CoV-2 virus detection is currently regarded as the gold standard (2, 41, 42), carefully performed future studies need to be carried out to determine the relative diagnostic accuracies and specificities of the saliva and NPS tests.

At present only two studies have considered the specificity of the saliva tests. In one of those tests only one saliva sample was found to be positive among 50 apparently healthy individuals who were PCR-negative for the NPS test (18). In the other work two individuals were detected positive in saliva tests on 98 participants who were negative for NPS test (21). These results may reflect a real difference in the specificities of the NPS and saliva tests, or they may simply be a consequence of occasional false negatives in the NPS tests.

For optimal saliva-based testing at least three conditions have to be improved by standardization and validation (43). (1) A specific saliva collection method should be selected and optimized after systematically comparing the various methods currently used for collecting whole saliva in other clinical and scientific contexts. (2) The optimal solution for collecting, transporting and storing saliva samples should be found. (3) The RNA assay method, either RT-qPCR, loop-mediated isothermal amplification (LAMP) or another protocol, should also be optimized for saliva, using an appropriate internal control; this cannot be human DNA which is overwhelming in NPS but not in saliva samples (15–18, 21). In order to obtain a reliable and sensitive saliva test, all of these conditions must be standardized.

Not surprisingly the studies included in our analysis used different sampling methods to collect saliva. This may have had a significant effect on the sensitivity of the saliva test. Azzi et al. used a simple drooling technique to collect saliva and they resuspended the collected specimens in 2 ml of PBS (14). In contrast, To et al. collected saliva specimens that also contained fluid from the posterior oropharynx obtained by coughing up and clearing the throat (17). Another study (18) asked patients to pool saliva in their mouth prior to collection, and to spit 1–2 ml into a collection pot. The act of pooling saliva in the mouth may have stimulated additional saliva secretion, which could have diluted the viral load in the specimen. In this case no transport medium was added to the specimens but, after transportation to the laboratories, liquid Amies medium was added. Wyllie et al. used a self-collection technique: patients were asked to spit repeatedly into a sterile urine cup until one third was full (21). This too could have diluted the sample with additional virus-free saliva. The remaining two studies did not describe the collection method at all (15, 16). Additionally, two of the studies specified that specimens were collected in early morning to avoid anomalies introduced by eating, drinking and tooth brushing (17, 21). The rest of the studies did not specify the time of collection or mention any other confounding factors that may have affected the sample. Taken together, the sample collection protocols of the included studies are quite diverse. But it is promising that even without validated, standardized collection protocols, the studies reviewed here yielded very similar results.

Other factors, such as the type of transport medium, the temperature during transportation, and the time passed between specimen collection and RNA extraction, may also affect the outcome of the tests (43). Unfortunately, there is insufficient information in these few studies to draw any conclusions about the possible effects of these confounding factors on the accuracy of saliva testing for COVID-19 diagnosis (15–18, 21). But again, although the five studies used different RNA isolation methods, and different PCR primers and conditions, it is encouraging to note that the virus could in all cases be detected in saliva samples with a consistently high level of sensitivity.

It is likely that a simple drooling technique, with no specific target volume and no extra stimulation of saliva secretion, will provide the greatest sensitivity if the viral RNA in whole saliva derives mainly from sources other than the secretions of the salivary glands. Drooling is a well-established saliva collection method that is generally recommended for analytical purposes (44). Due to its simplicity, it does not require trained personnel, it can be self-administered, and it can be done at home if necessary. Even in the clinic, the drooling method is safer than nasopharyngeal or oropharyngeal swabbing, with no need for infected swabs to be carried through the air from the patient to the container. The fact that nasopharyngeal swab sampling sometimes has to be repeated in overt COVID-19 patients before a positive result is obtained suggests that the reliability of that sampling method is lower than might be expected from saliva sampling. Moreover, this saliva collecting technique also avoids the mixing of fluids from different anatomical regions such as the oropharynx (14).

In the present meta-analysis the overall sensitivity of the saliva (index) test is assessed by comparison with the NPS (reference standard) test using patient-based pooled data. This simple comparison does not allow us to address any of the more complex questions that arise from the widely varying presentation of different COVID-19 patients. For example, are there significant differences in the sensitivities of the two sampling methods according to the primary location of the infected cells? Are there higher viral loads in the saliva, and is there therefore a higher saliva test sensitivity, in COVID-19 patients who only present with a loss of taste sensation or who are asymptomatic? Are saliva tests more or less sensitive than NPS tests in patients whose infection is mainly localized to the respiratory tract? Correlation studies comparing saliva and NPS viral loads in patients categorized by the nature and severity of their symptoms should be very informative. Time series data on the relative viral loads in the saliva and NPS specimens may be useful in predicting the progression of the disease and in guiding treatment. But, as discussed above, these studies will require careful optimization and standardization, particularly of the saliva collection protocol.

The need for reliable, non-invasive and easy-to-perform tests for COVID-19 has focused special attention on saliva in the last few months. Between 1 January and 25 April 2020, 18 clinical trials involving saliva specimens have started according to the ClinicalTrials.gov registry (Table S2). Among these, 13 are non-interventional, focusing on the diagnostic value of various specimens including saliva, and five interventional studies also planned to use saliva as a diagnostic tool, but with a primary focus on evaluating potential treatments for SARS-CoV-2 infections. Unfortunately, these registered clinical trials vary considerably in the amount of information presented about the proposed testing methodology. Neither the non-interventional nor the interventional protocols have clear descriptions of the collection, transportation and storage of saliva samples, and the optimization of the viral RNA assay for saliva specimens. Only a few of them emphasize the necessity for determining the sensitivity and specificity of the saliva-based test. But hopefully, during the course of execution, such studies will yield high quality, reliable data that can be used to address some of the important biological and methodological questions that we have discussed here.

## Limitations

A limitation of the present work is the relatively small number of studies and small sample sizes available regarding this topic. Despite the large number of records found in the systematic search of the literature, only 6 studies could be included. Although intensive research is in progress regarding COVID-19, there are still only a handful articles fulfilling our eligibility criteria. The limited amount of reported data makes it difficult to perform comprehensive analyses and to thoroughly investigate the causes behind certain trends in the results. Another issue that hinders in-depth analysis is the lack of methodological homogeneity, and the inadequate reporting of methods and outcome parameters. A significant limitation is the lack of false-positive data, based on an independent reference, that would be required for 2 × 2 contingency tables to allow estimation of the test specificities. Thus, the more rigorous statistical methodologies specially developed for meta-analysis of diagnostic test accuracy could not be used in this work.

All studies except two (18, 21) investigated the reliability of the saliva test only among confirmed COVID-19 infected participants, with no healthy individuals or asymptomatic COVID-19 patients recruited for comparison. Additionally, there are several other confounding factors that might have affected the detectability of viral RNA in the saliva, such as the timing and method of sample collection, the choice of transport medium, storage and transport temperatures, the time passed between specimen collection and RNA isolation, and the extraction and PCR kits used for isolation, amplification and detection. None of these factors could be properly addressed in our analysis owing to the lack of information in the reported studies.

## Conclusion

In the present meta-analysis we provide evidence that saliva tests are a promising alternative to nasopharyngeal swab tests for COVID-19 diagnosis. Optimized and validated saliva assays offer the possibility of reliable self-collection of samples for COVID-19 testing in the future. However, there are many open questions to be answered before the precise specificity and sensitivity of the saliva-based tests can be determined and appropriate standardized procedures introduced into clinical practice.

## Data Availability Statement

Publicly available datasets were analyzed in this study. This data can be found here: The data that support the findings of this study are available from the corresponding author, upon reasonable request.

## Author Contributions

LC, SK, ÁN, ZL, ZS, PH, MS, and GV devised the project, the main conceptual ideas and planned the research. LC, SK, NF, ZS, and GV worked out the methodology. IM, AH, and LC performed the data collection, literature search, study selection, and data extraction. LC, IM, and AH also organized and maintained research data for analysis. NF performed analytic calculations and applied statistical models for synthetizing data. ZS and SK also aided the research by interpretation of raw and synthetized data. NF visualized synthetized data into forest plots. LC and GV worked on summarizing results into figures and tables. SK and GV were responsible for managing and coordinating the research activity. PH and GV took leadership responsibility for the research activity, provided resources, and acquired financial support for the research project. ZS, PH, MS, and GV validated reproducibility of the results. LC, NF, IM, AH, and GV wrote the manuscript with input from all authors. SK, ÁN, ZL, ZS, PH, MS, and GV extensively reviewed the work and further edited the manuscript. All authors contributed to the article and approved the submitted version.

## Conflict of Interest

The authors declare that the research was conducted in the absence of any commercial or financial relationships that could be construed as a potential conflict of interest.

## Supplementary Material

Table S1 PRISMA checklist.

Table S2 Characteristics of clinical trials including saliva as a diagnostic tool for COVID-19, registered on ClinicalTrials.gov.

Table S3 Summary of risk-of-bias and applicability concerns in included studies.

Table S4 Detailed summary of risk of bias and applicability across studies.
