# Supplementary material for: Saliva as a Candidate for COVID-19 Diagnostic Testing: A Meta-Analysis
Source: Front Med (Lausanne). 2020 Aug 4;7:465. doi: 10.3389/fmed.2020.00465 (PMC7438940; doi:10.3389/fmed.2020.00465)
Supplement: Table S2 — Characteristics of clinical trials including saliva as a diagnostic tool for COVID-19, registered on ClinicalTrials.gov. [file Table_2.DOCX]

**Table S2**. Characteristics of clinical trials including saliva as a diagnostic tool for COVID-19, registered on ClinicalTrials.gov

| **ID** | NCT04332107 | NCT04321174 | NCT04352959 | NCT04354259 | NCT04276688 |
| --- | --- | --- | --- | --- | --- |
| **Recruiting Status** | Recruiting | Recruiting | Recruiting | Recruiting | Completed |
| **Study type** | interventional | interventional | interventional | interventional | interventional |
| **Number of Centers and Study Design** | Single center, interventional, randomized (RCT), parallel assignment, quadruple masking, phase 3 | Multi-locations, interventional, randomized (RCT), parallel assignment, single masking (outcomes assessor),  phase 3 | Multi-locations, interventional, randomized (RCT), parallel assignment, triple masking | Single center, interventional, randomized (RCT), parallel assignment, none masking,  phase 2 | Single center, interventional, randomized (RCT), parallel assignment, none masked,  Phase 2 |
| **Location** | the USA | Canada | France | Canada | China |
| **Population** | Subjects with positive SARS-CoV-2 test results received within the previous three days, but not hospitalized  (n=2271) | 1) High risk close contact with a confirmed COVID-19 case during their symptomatic period, 2) Successfully contacted by the study team within 24 hours of study team notification of the relevant index COVID-19 case  (n=1220) | Patients with clinical diagnosis of Covid-19 infection (n=178) | 1) For ambulatory cohort: patients confirmed COVID-19 infection by PCR within 5 days of symptom onset discharged to home isolation,  2) For hospitalized cohort: SARS-CoV-2 RNA-positive on nasopharyngeal swab / respiratory specimen within 5 days of symptom onset admitted to hospital for management of COVID-19  (n=140) | Subjects include patients hospitalized for confirmed 2019-n-CoV infection, temperature ≥38°C with another symptoms upon admission  (n=127) |
| **Intervention** | Single oral 1g dose of Azythromycin | Lopinavir/Ritonavir 400/100 mg twice daily for 14 days | Mouthrinse with bêta-cyclodextrin and citrox 3 daily mouthrinses for 7 days | Single dose of peginterferon lambda 180µg sc at baseline for ambulatory cohort and peginterferon lambda 180µg sc at baseline and a second dose on day 7 for hospitalized cohort | Lopinavir/Ritonavir 400/100 mg twice daily for 14 days, Ribavirin 400 mg twice daily for 14 days and IFN-beta-1B 0.25 mg sc injection alternate day for 3 day / Nasopharyngeal swab, saliva, urine, stool and blood sampling |
| **Comparison** | placebo | no intervention | Placebo: mouth rinse without antiviral | No specific therapy for ambulatory cohort and the best supportive care for hospitalized cohort | Lopinavir/Ritonavir 400/100 mg twice daily for 14 days |
| **Primary Outcomes** | All-cause hospitalization or emergency room stay of >24 hours | The primary outcome is microbiologically confirmed COVID-19 infection, ie. detection of viral RNA in a respiratory specimen (mid-turbinate swab, nasopharyngeal swab, sputum specimen, saliva specimen, oral swab, endotracheal aspirate, bronchoalveolar lavage specimen) by day 14 of the study. | Change from baseline amount of SARS-CoV-2 in salivary samples at 7 days | 1) The proportion of participants with negative SARS-CoV-2 RNA on nasopharyngeal swab, Nasopharyngeal swab, saliva and blood sampling. 2) Rate of combined treatment-emergent and treatment-related severe adverse events | Time to negative nasopharyngeal swab |
| **Secondary outcome** | viral load by self-collected nasal swab, Viral load by self-collected saliva swab | - | Change from Baseline amount of SARS-CoV-2 virus in nasal samples at 7 days | - | Time to negative saliva 2019-n-CoV RT-PCR |

**Table S2.** Continued

| **ID** | NCT04360811 | NCT04354610 | NCT04361604 | NCT04325919 | NCT04351646 |
| --- | --- | --- | --- | --- | --- |
| **Recruiting Status** | Recruiting | Recruiting | Not yet recruiting | Recruiting | Recruiting |
| **Study type** | non-interventional | non-interventional | non-interventional | non-interventional | non-interventional |
| **Number of Centers and Study Design** | Single center, observational, non-randomized (NRCT), parallel assignment, none masked | Multi-locations, observational, single group assignment, none masked | Single center, observational, cohort, prospective | Observational | Single center, observational, case-control, prospective |
| **Location** | France | France | France | China | UK |
| **Population** | 1) Unexposed group: COVID 19 negative pregnant woman, 2) Exposed group: COVID 19 positive (symptomatic and asymptomatic) pregnant woman  (n=3600) | Patients hospitalized for critical form of Covid-19 infection within 3 days  (n=57) | 1) Patients co infected HIV and SRAS-CoV2 (n=250), 2) Patients infected HIV without COVID-19 (n=20) | Patients with laboratory-confirmed COVID-19, (n=170) Patients hospitalized for pneumonia tested negative for COVID-19 are controls | 1) SARS-CoV-2 negative inpatients,  2) SARS-CoV-2 positive inpatients,  3) SARS-CoV-2 suspected or confirmed SARS-CoV-2 positive cases amongst health care professionals and lab staff  (n=500) |
| **Intervention** | N/A | N/A | N/A | N/A | N/A |
| **Comparison** | N/A | N/A | N/A | N/A | N/A |
| **Primary Outcomes** | Exposure to SARS-CoV-2 will be measured the day of delivery by RT-PCR on maternal saliva and by serology on maternal blood | 1) Worsening of renal function by at least KDIGO grade 1 during hospitalization for Covid-19 infection, 2) Troponin greater than 99th percentile during hospitalization for Covid-19 infection | Describe the course of COVID-19 disease in patients infected with HIV, biological sampling (blood, saliva, rectal swab (stool swab), urine, nasopharyngeal swab, conjonctival swab, semen | Patients' treatment and management during hospitalization. Serial viral load changes during hospitalization. Collection of blood, stool, rectal swab, urine, saliva, nasopharyngeal aspirate/flocked swab, sputum/tracheal aspirate | Antibody titres to SARS-CoV-2 at specified days post baseline samples (Nasopharyngeal swab, blood and saliva sampling) |
| **Secondary outcome** | Description of the number of positive COVID-19 RT-PCRs in the conception products: amniotic fluid, frozen placenta fragment, frozen fetal tissue, cord blood or frozen cord fragment | Blood samples, **saliva** collection, and urine collection to carry out biomarker assays and for the constitution of a biological collection. | - | - | - |

**Table S2**. Continued

| **ID** | NCT04337424 | NCT04357977 | NCT04356586 | NCT04355533 | NCT04362150 |
| --- | --- | --- | --- | --- | --- |
| **Recruiting Status** | Recruiting | Recruiting | Enrolling by invitation | Recruiting | Recruiting |
| **Study type** | non-interventional | non-interventional | non-interventional | non-interventional | non-interventional |
| **Number of Centers and Study Design** | Single center, observational, case-control, prospective | Multi-locations, observational, cross-sectional | Single center, observational, cohort, prospective | Single center, observational, non-randomized (NRCT), single group assignment, none masked | Single center, observational, cohort, prospective |
| **Location** | France | USA | Belgium | France | USA |
| **Population** | 1) Patients diagnosed positive,  2) Healthcare staff presumed negative for SARS-CoV-2 (n=180) | Patients and study staff at the testing site who have been flagged for COVID-19 testing or who are being treated for COVID-19 (n=300) | Healthcare workers with mild symptoms for Covid-19  (n=300) | Children hospitalized since at most 4 days and their parents  (n=1920) | Individuals with positive test for COVID-19 who have recovered from acute infection (wide spectrum of age, race, gender and disease severity) (n=800) |
| **Intervention** | N/A | N/A | N/A | N/A | N/A |
| **Comparison** | N/A | N/A | N/A | N/A | N/A |
| **Primary Outcomes** | Comparison of LAMP test with reference RT-PCR on viral detection (Saliva and nasopharyngeal swab sampling) | RBA-2 saliva monitoring device development. Nasopharyngeal swab and saliva sample. The comparison of the results obtained from the current testing methods will be used to calibrate machine learning algorithms of the RBA-2 | 1) Percentage of serological positive healthcare workers, 2) Percentage of healthcare workers with positive saliva swabs | Seroconversion against SARS-CoV2 in children, Nasopharyngeal, rectal swabs, saliva and blood sampling | Demographic data on participants and Proportion of participants previously hospitalized. Whole blood, peripheral blood mononuclear cells, plasma, serum and saliva. |
| **Secondary outcome** | **-** | - | - | - | - |

**Table S2**. Continued

| **ID** | NCT04357327 | NCT04336215 | NCT04348240 |
| --- | --- | --- | --- |
| **Recruiting Status** | Recruiting | Recruiting | Recruiting |
| **Study type** | non-interventional | non-interventional | non-interventional |
| **Number of Centers and Study Design** | Single center, non-randomized (RCT), parallel assignment, single masking | Multi-locations, observational, cohort, prospective | Single center, observational, cohort, prospective |
| **Location** | Italy | USA | USA |
| **Population** | 1) Patients with symptoms associated with COVID-19, 2) Asymptomatic patients with low risk phenotype (n=100) | 1) Healthcare workers (n=500), 2) Non-healthcare workers: faculty staff and students, who do not have patient contact (n=250), 3) Multigenerational household members, who test positive and negative for SARS-CoV-2  (n=540) | 1) Asymptomatic high-risk subjects with known history of close personal contact with a COVID-19 positive person not tested (SARS-CoV2 status unknown), 2) Asymptomatic or mildly symptomatic subjects who are COVID-19 positive, 3) COVID-19 positive individuals retesting negative (n=60) |
| **Intervention** | N/A | N/A | N/A |
| **Comparison** | N/A | N/A | N/A |
| **Primary Outcomes** | 1) Sensibility after 10 minutes for salivary test and after 6 hours for the nasopharyngeal swab, 2) Specificity after 10 minutes for salivary test and after 6 hours for the nasopharyngeal swab | 1) Prevalence, 2) Incidence, Nasopharyngeal swab, saliva and blood sampling | Determination of SARS-CoV-2 viral load and infectivity in saliva that may contribute to asymptomatic transmission. Collection of nasal and oral secretions and droplets produced by participants while they speak |
| **Secondary outcome** | **-** | - | - |
