# Supplementary material for: Saliva as a Candidate for COVID-19 Diagnostic Testing: A Meta-Analysis
Source: Front Med (Lausanne). 2020 Aug 4;7:465. doi: 10.3389/fmed.2020.00465 (PMC7438940; doi:10.3389/fmed.2020.00465)
Supplement: Table S3 — Summary of risk-of-bias and applicability concerns in included studies. [file Table_3.DOCX]

**Table S3**. Summary of risk-of-bias and applicability concerns in included studies.

| **STUDY** | **RISK OF BIAS** | | | |  | **APPLICABILITY CONCERNS** | | |
| --- | --- | --- | --- | --- | --- | --- | --- | --- |
|  | **PATIENT SELECTION** | **INDEX**  **TEST** | **REFERENCE STANDARD** | **FLOW AND TIMING** |  | **PATIENT SELECTION** | **INDEX**  **TEST** | **REFERENCE STANDARD** |
| **Azzi et al. (2020)** | **✓** | **✗** | **✓** | **✗** |  | **✓** | **✓** | **✓** |
| **Bae et al. (2020)** | **✓** | **✗** | **✓** | **?** |  | **✓** | **?** | **?** |
| **Fang et al. (2020)** | **✓** | **✗** | **✓** | **?** |  | **✓** | **?** | **✓** |
| **To et al. (2020)** | **✓** | **✗** | **?** | **?** |  | **✓** | **✓** | **✓** |
| **Williams et al. (2020)** | **?** | **?** | **?** | **✗** |  | **✓** | **✓** | **?** |
|  |  |  |  |  |  |  |  |  |
| **Not included in the quantitative analysis:** | | |  |  |  |  |  |  |
| **Wyllie et al. (2020)** | **✓** | **?** | **?** | **?** |  | **✓** | **✓** | **✓** |

**✓** = Low Risk **✗**= High Risk **?** = Unclear Risk
