# Supplementary material for: Saliva as a Candidate for COVID-19 Diagnostic Testing: A Meta-Analysis
Source: Front Med (Lausanne). 2020 Aug 4;7:465. doi: 10.3389/fmed.2020.00465 (PMC7438940; doi:10.3389/fmed.2020.00465)
Supplement: Table S4 — Detailed summary of risk of bias and applicability across studies. [file Table_4.DOCX]

**Table S4**. Detailed summary of risk of bias and applicability across studies.

| **Risk of bias** | **Yes** | **No** | **Unclear** |
| --- | --- | --- | --- |
| **DOMAIN 1: PATIENT SELECTION** | | | |
| Was a consecutive or random sample of patients enrolled? | 6 | 0 | 0 |
| Was a case-control design avoided? | N/A | | |
| Did the study avoid inappropriate exclusions? | 5 | 0 | 1 |
| **Could the selection of patients have introduced bias?** | **5** | **0** | **1** |
| **DOMAIN 2: INDEX TEST(S)** | | | |
| Were the index test results interpreted without knowledge of the results of the reference standard? | 0 | 4 | 2 |
| If a threshold was used, was it pre-specified? | 0 | 0 | 6 |
| **Could the conduct or interpretation of the index test have introduced bias?** | **0** | **4** | **2** |
| **DOMAIN 3: REFERENCE STANDARD** | | | |
| Is the reference standard likely to correctly classify the target condition? | 4 | 0 | 2 |
| Were the reference standard results interpreted without knowledge of the results of the index test? | 3 | 0 | 3 |
| **Could the reference standard, its conduct, or its interpretation have introduced bias?** | **3** | **0** | **3** |
| **DOMAIN 4: FLOW AND TIMING** | | | |
| Did all patients receive a reference standard? | 6 | 0 | 0 |
| Did patients receive the same reference standard? | 6 | 0 | 0 |
| Were all patients included in the analysis? | 5 | 1 | 0 |
| **Could the patient flow have introduced bias?** | **0** | **2** | **4** |
|  | | | |
| **Applicability concerns** | **Low** | **High** | **Unclear** |
| **DOMAIN 1: PATIENT SELECTION** | | | |
| **Is there concern that the included patients do not match the review question?** | **6** | **0** | **0** |
| **DOMAIN 2: INDEX TEST(S)** |  |  |  |
| **Is there concern that the index test, its conduct, or interpretation differ from the review question?** | **4** | **0** | **2** |
| **DOMAIN 3: REFERENCE STANDARD** |  |  |  |
| **Is there concern that the target condition as defined by the reference standard does not match the review question?** | **4** | **0** | **2** |
